# The All-on-four concept for fixed full-arch rehabilitation of the edentulous maxilla and mandible: a longitudinal study in Japanese patients with 3–17-year follow-up and analysis of risk factors for survival rate

**DOI:** 10.1186/s40729-023-00511-0

**Published:** 2023-11-08

**Authors:** Takashi Uesugi, Yoshiaki Shimoo, Motohiro Munakata, Daisuke Sato, Kikue Yamaguchi, Michiya Fujimaki, Kazuhisa Nakayama, Tae Watanabe, Paulo Malo

**Affiliations:** 1 Malo Dental and Medical Tokyo, Fukuhara Ginza 8F, 7-8-10 Ginza, Chuo-Ku, Tokyo, 104-0061 Japan; 2 Department of Implant Dentistry, Showa University School of Dentistry, Tokyo, Japan

**Keywords:** All-on-four, Immediate loading, Implant failure, Survival rate, Edentulous

## Abstract

**Purpose:**

Implant-supported immediately loaded fixed full-arch rehabilitation via All-on-four treatment yields good long-term results for both the maxilla and the mandible. However, the risk factors affecting long-term implant survival are unknown, and the long-term prognosis of All-on-four concept procedures in Japanese individuals has not been elucidated. We aimed to determine the cumulative implant survival rate after 3–17-year follow-up and identify the associated risk factors.

**Methods:**

We analysed 561 cases (307 maxillae, 254 mandibles) with 2364 implants (1324 maxillae, 1040 mandibles) that received All-on-four treatment. We investigated the cumulative implant- and patient-level survival rates and various risk factors for implant failure. Statistical analysis was performed using the log-rank test for differences in Kaplan–Meier curves, univariate analysis using the Chi-square test, and multivariate analysis for risk factors affecting the survival rate.

**Results:**

The cumulative survival rate was 94.4% by patient level and 97.4% by implant level for the maxilla, and 96.7% by patient level and 98.9% by implant for the mandible, with up to 17 years of follow-up. The maxillary survival rate at the implant level was significantly lower (*p* < 0.05). Furthermore, the maxillary survival rate within 24 months was significantly lower at the implant level (*p* < 0.01). Multivariate analysis revealed that the maxilla was the most significant risk factor (*p* < 0.01).

**Conclusions:**

All-on-four treatment yielded high long-term survival rates in Japanese patients. However, the maxilla showed a significantly lower cumulative survival rate than the mandible, while early failure was significantly higher. Furthermore, the maxilla was a significant risk factor influencing the survival rate.

## Background

For edentulous or dentulous patients with severe, full-arch periodontitis that makes preservation difficult, Malo et al. avoided guided bone regeneration. Instead, they performed implant insertion, devising what is now known as the All-on-four concept, in which immediate loading is performed. They reported mandibular cases treated with the procedure in 2003 [1] and maxillary cases in 2005 [2]. Although there are differences in the survival rates of the maxilla and mandible depending on the follow-up period, recent clinical studies have also reported implant survival rates of 94.7% (5–13 years) in the maxilla and 93% (10–18 years) in the mandible, indicating favourable long-term progress [3, 4]. Furthermore, in a comparison of immediate loading and delayed loading for fixed full-arch rehabilitation, the 10-year survival rate was 93.3% for immediate loading and 94.9% for delayed loading. That is, full-arch immediate load treatment based on the All-on-four concept has been established as a reliable option for fixed prosthesis placement in edentulous patients for both the maxilla and mandible [5]. However, there have been reports of early implant failure due to immediate loading and subsequent damage/breakage of prosthetic devices [5–8]. A systematic review also reported that although there was no difference between the survival rates of immediate loading and conventional loading, the failure rate of implants was high (risk ratio = 1.92) [9]. Furthermore, to date, there have been few studies on risk factors that affect the survival rate of immediate-loaded full-arch implants. Moreover, there are no reports on the long-term prognosis of All-on-four concept treatment in Japanese individuals. Thus, in this study, we treated the maxilla and mandible with the All-on-four concept with the aim of investigating the cumulative implant survival rate after 3–17 years and examining the risk factors that affect the survival rate.

## Methods

### Study population

In this study, we enrolled patients who underwent tooth extraction, All-on-four concept-based implant insertion and immediately loaded fixed full-arch denture fitting at a private rehabilitation centre (Malo Dental and Medical Tokyo, Tokyo, Japan) as a treatment for edentulous jaws or partially edentulous jaws with remaining teeth in poor condition between September 2005 and March 2019.

The exclusion criteria were patients who did not undergo follow-up at the private practice and those with zygomatic implants.

This study was approved by the ethics committee for research involving human subjects (ethics review committee number 11000688 approval, approval number 21-055-A), and all patients provided written informed consent for study inclusion.

### Surgical protocol

**Fig. 1:**
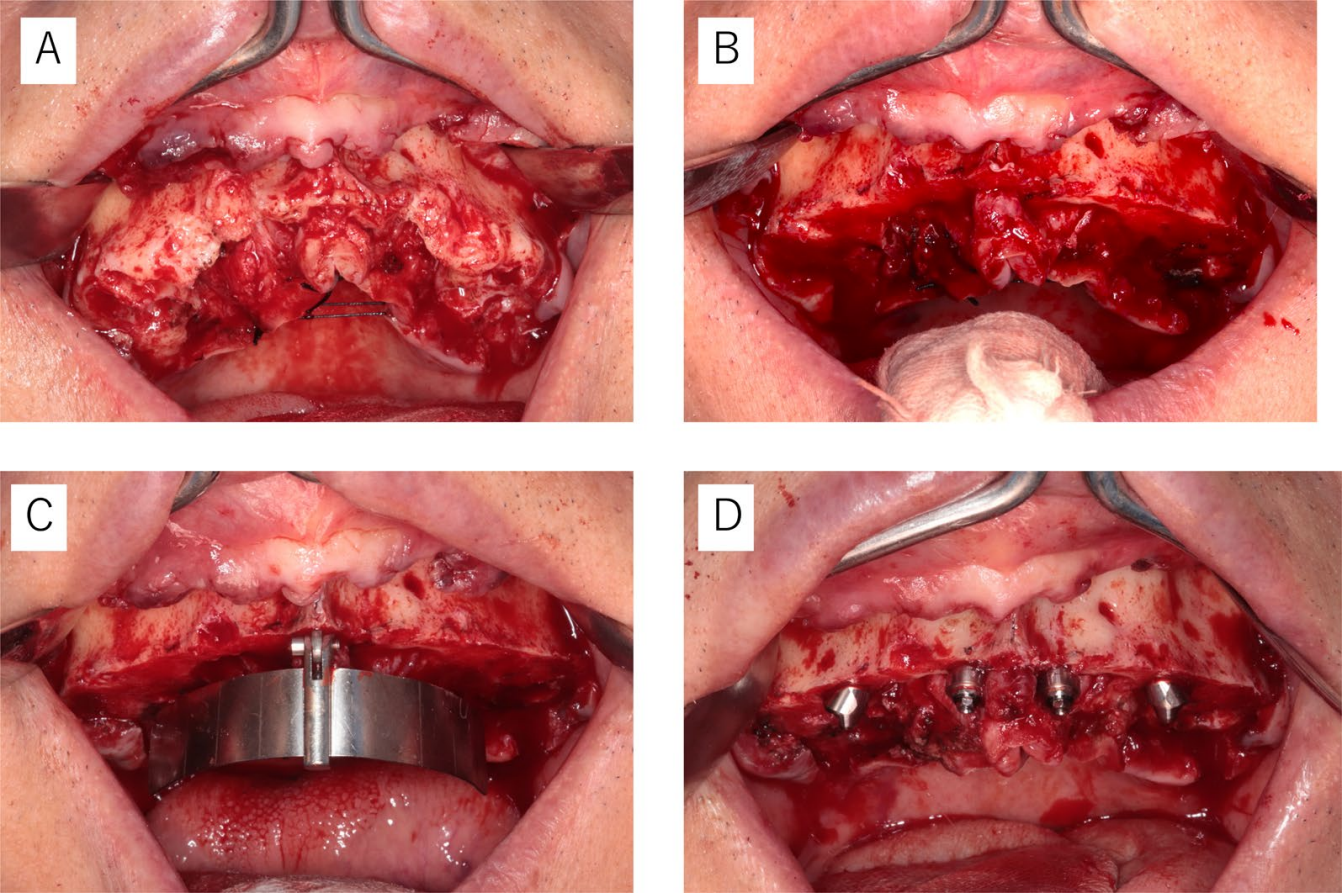
Surgical protocol. **a** After extraction tooth. **b** After bone reduction. **c** Placed the All-on-4 Guide. **d** Placed implants and abutment

The surgical procedures for the All-on-four concept were performed in accordance with those detailed previously by Malo et al. [3, 4] Surgery was performed under local infiltration anaesthesia (2% lidocaine, including 1/80,000 adrenaline). The surgical protocol is outlined below and in Fig. 1:

1. In patients with remaining teeth, these teeth are extracted.
2. A longitudinal incision is made on the mucosa distal to the first molars on both sides, and a transverse incision is made on the mucosa on the alveolar crest slightly on the lingual/palatal side to form a mucoperiosteal flap.
3. If necessary, shaping of the alveolar bone and jawbone is performed for the purpose of securing the clearance required for prosthetic device fabrication and levelling the base surface of the prosthetic device.
4. For the maxillary sinus, a portion of the anterior wall of the maxillary sinus is excised using a round-burr tip, and a probe is used to explore the same area to confirm the morphology of the anterior maxillary sinus. For the mandible, the mental foramen is clearly indicated, and a probe is inserted in the mesial direction along the bone surface and used to confirm the nerve running morphology.
5. Insertion of the implant starts from the posterior end on both sides. The posterior implant should have a diameter of ≥ 4.0 mm. The implantation position and tilt angle are determined using a standardized surgical guide (All-on-four Guide, Nobel Biocare AG, Kloten, Switzerland).
6. The leading tip of the implant embedded in the posterior slope should be placed in the region of the mouth equivalent to the canines. Therefore, care should be taken not to cause interference, and the anterior implant should be placed in the area corresponding to the middle and lateral incisors. The anterior implant should have a diameter of ≥ 3.3 mm.
7. In principle, four implant bodies are inserted. However, if it is not possible to place an implant with a length of ≥ 10 mm, or if an initial fixation of ≥ 35 Ncm cannot be obtained, additional implants should be placed nearby if necessary and possible.
8. When inserting the implant, attach a straight or 17° angled abutment anteriorly and a 30° angled abutment posteriorly and suture them.

To prevent postoperative infection, patients received amoxicillin 250 mg four times daily for 5 days and 0.2% benzethonium chloride mouthwash four times a day for 2 weeks, as well as loxoprofen sodium 60 mg three times a day for 5 days as an analgesic. Sutures were removed 2 weeks postoperatively.

### Prosthetic protocol

**Fig. 2:**
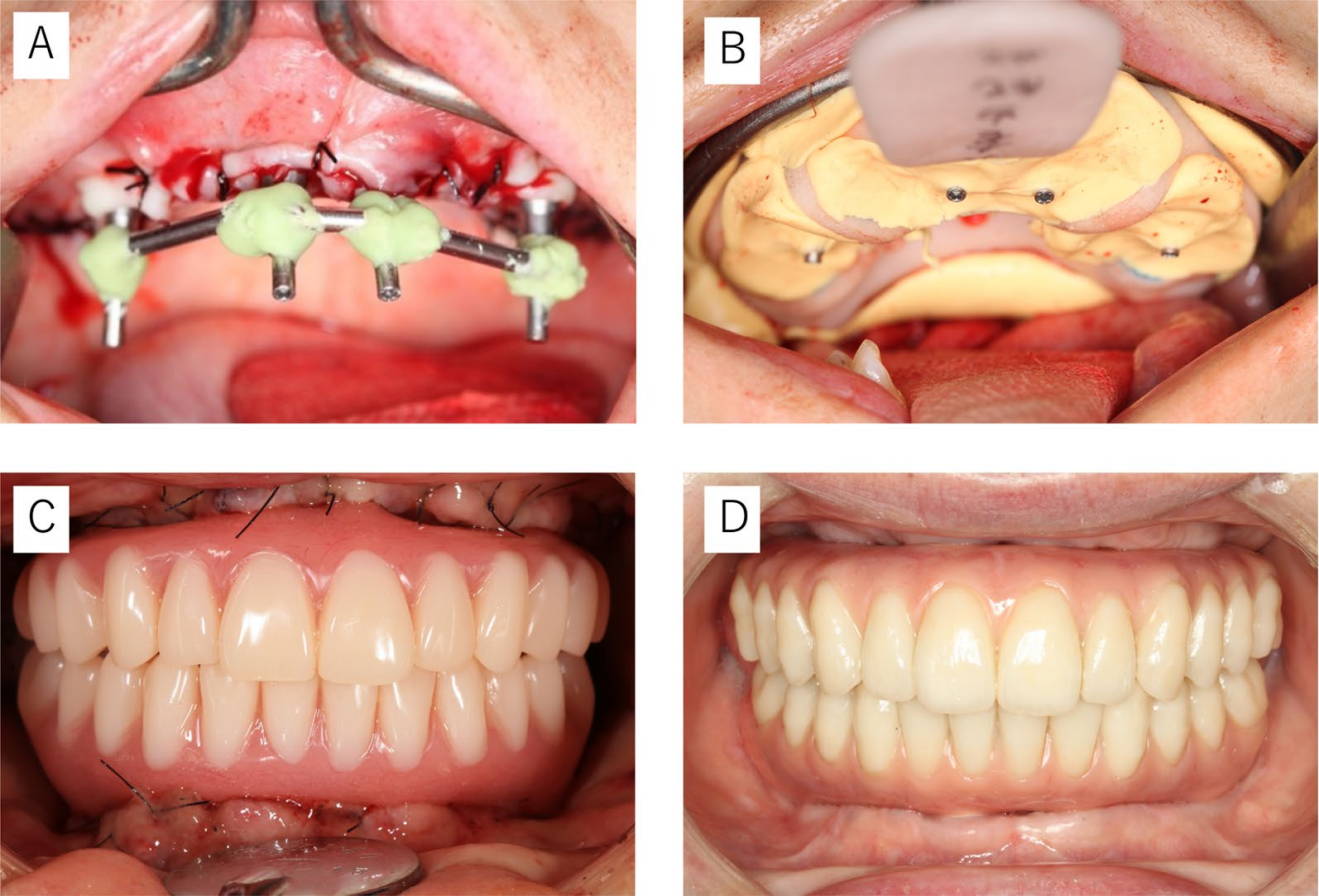
Prosthetic protocol. **a** Pretreatment of impression (connected to each impression coping). **b** Impression. **c** Immediate loading with provisional restoration after operation. **d** Final restoration

The prosthetic protocol is outlined in Fig. 2. Briefly, copings for open-tray impressions were connected to form a device with which impressions and bite registrations were taken. Using the indirect method, a temporary prosthesis was fabricated by inserting reinforcement wires cast from a Co–Cr alloy into a titanium temporary cylinder and acrylic resin (PROVISTA, Sun Medical Co., Ltd., Shiga, Japan). This prosthesis was attached to the patient on the same day.

The final prosthesis was fabricated 6 months after the provisional prosthesis. The final prosthesis was either fabricated as a titanium framework with ceramic crowns (IPS e.max Press, Ivoclar Vivadent, Schaan, Liechtenstein) or with acrylic resin crowns (anterior teeth [Bioblend, Dentsply Sirona, Bensheim, Germany] and posterior teeth [LIVDENT GRACE, GC Co., Tokyo, Japan]).

### Follow-up and maintenance protocol

The patients were instructed to maintain a soft-food diet for 2–3 months postoperatively. Follow-up clinical appointments were performed at 7 days, 14 days, and 1, 2, 3, 4, 5, and 6 months. After wearing the final prosthesis, follow-up was performed every 3–6 months, during which clinical parameters were evaluated, and oral hygiene instructions were given.

### Clinical outcomes

We evaluated the following clinical outcomes:

1. 3–17-year cumulative survival rate (implant and patient levels): implant survival criteria were classified as failure if the implants were removed due to movement and inflammatory symptoms, such as persistent pain, swelling, and abscesses. For patient-level cumulative survival, failure was defined as the loss of one or more of the implants in a patient.
2. Patient profile of implant failure and early implant failure
3. Investigation of risk factors (implant-related and patient-related factors) related to survival rate
4. Implant-related factors: type of implant, implant length, primary stability, and angle of implant placement.
5. Patient-related factors: sex, systemic disease, smoking habit (number of cigarettes smoked, ≥ 10 cigarettes/day), and treatment area (maxilla/mandible).

### Statistical analysis

The cumulative survival rate was analysed using the Kaplan–Meier method, and comparisons of survival rates between groups were performed using the log-rank/Wilcoxon test. The various risk factors influencing implant survival rate (implant failure) were analysed univariately using the Chi-square test, followed by multivariate analysis using logistic regression analysis. In addition, odds ratios (OR) were calculated for the risk factors. All statistical analyses were performed using IBM SPSS Statistics 20 for Windows (International Business Machines Corp, Armonk, NY, USA). *p* < 0.05 was considered to indicate statistical significance.

## Results

Patient and implant data and characteristics are shown in Tables 1, 2, 3.

**Table 1:** Descriptive patient data

	Number
Total	561
Sex	
Male	287
Female	274
Age (years; mean ± SD)	57.2 ± 10.4
Observation period (months; mean ± SD)	105.7 ± 44.5
Smoker	
Yes	200
No	361
Systemic disease	
Healthy	332
Diabetes	19
Osteoporosis	4
Circulatory diseases	93
Combined*	14
Other**	99

*Combined: systemic disease combination of diabetes and circulatory disease (*n* = 13); osteoporosis and circulatory disease (*n* = 1)

**For example, dyslipidemia, asthma, thyroid disease

**Table 2:** Descriptive implant data

	Number
Implants	
Total	2364
Maxilla	1324
Mandibula	1040
Implant systems	
Nobel speedy groovy	1925
Straumann bone level tapered	278
Nobel parallel CC	52
Nobel replace tapered groovy	50
Brånemark system Mk IV TiUnite	32
Nobel replace tapered	23
Nobel active	4
Implant angulation	
Straight	1242
Tilted	1122
Implant length (mm)	
7	20
8	8
8.5	26
10	111
11.5	100
12	59
13	292
14	53
15	564
16	135
18	802
20	127
22	60
25	7

**Table 3:** Patient and implant-related characteristics

Patient-related	*n* = 561 (%)
Sex	
Male	*n* = 287 (51.2)
Female	*n* = 274 (48.8)
Age at placement, mean (SD)	57.2 (10.4)
Observation period, mean (SD)	105.7 (44.5)
Smoker	
Yes	*n* = 200 (35.7)
No	*n* = 361 (64.3)
Systemic disease	
Healthy	*n* = 332 (59.2)
Diabetes	*n* = 19 (3.4)
Osteoporosis	*n* = 4 (0.7)
Circulatory diseases	*n* = 93 (16.6)
Combined*	*n* = 14 (2.5)
Other**	*n* = 99 (17.6)

Implant-related	*n* = 2364 (%)
Jaw	
Maxilla	1324 (56.0)
Mandibula	1040 (44.0)
Implant systems	
Nobel speedy groovy	1925 (81.4)
Straumann bone level tapered	278 (11.8)
Nobel parallel CC	52 (2.2)
Nobel replace tapered groovy	50 (2.1)
Brånemark system Mk IV TiUnite	32 (1.4)
Nobel replace tapered	23 (1.0)
Nobel active	4 (0.2)
Implant angulation	
Straight	1242 (52.5)
Tilted	1122 (47.5)
Implant length (mm)	
< 10 mm	54 (2.3)
10 mm ≤ , < 15 mm	615 (26.0)
15 mm ≤ , < 18 mm	699 (29.6)
18 mm ≤	996 (42.1)

*Combined: systemic disease combination of diabetes and circulatory disease (*n* = 13); osteoporosis and circulatory disease (*n* = 1)

**For example, dyslipidemia, asthma, thyroid disease

A total of 561 patients (307 maxillae, 254 mandibles) and 2364 implants (1324 maxillae, 1040 mandibles) were included. Maxillary cases included 156 males and 151 females, with an average age of 57.2 ± 10.4 years and an average follow-up period of 105.7 ± 44.5 months. Mandibular cases included 131 males and 123 females, with an average age of 55.1 ± 10.6 years and an average follow-up period of 108.3 ± 42.6 months.

### Cumulative implant survival rate

The number of failed implants was 22 and 7 in the maxilla and mandible, respectively, and the number of patients was 11 and 6 in the maxilla and mandible, respectively. The number of implants and patients who failed within 24 months were 19 implants (0–12 months: 15 implants, 12–24 months: four implants), nine patients (0–12 months: eight patients, 12–24 months: one patient) in the maxilla and three implants (breakdown; 0–12 months: three implants, 12–24 months: 0 implants), three patients (breakdown; 0–12 months: three patients, 12–24 months: 0 patients) in the mandible. Furthermore, the number of implants that failed at ≥ 24 months and the number of patients were as follows: three implants (breakdown; 24–36 months: one implant, 168–180 months: two implants), two patients (breakdown; 24–36 months: one patient, 168–180 months: one patient) in the maxilla and four implants (breakdown; 24–36 months: one implant, 72–84 months: one implant, 132–144 months: two implants), three patients (breakdown; 24–36 months: one patient, 72–84 months: one patient, 132–144 months: one patient) in the mandible (Tables 4, 5, 6, 7).

**Table 4:** Survival rate and number of implants depending on the investigation interval (implant level in the maxilla)

Investigation interval (months)	Number of implants	Failed implants	Survival rate intervals (%)
0 < , ≤ 12	1324	15	100–98.9
12 < , ≤ 24	1309	4	98.9–98.6
24 < , ≤ 36	1305	1	98.6–98.5
36 < , ≤ 48	1304	0	98.5
48 < , ≤ 60	1222	0	98.5
60 < , ≤ 72	1116	0	98.5
72 < , ≤ 84	978	0	98.5
84 < , ≤ 96	835	0	98.5
96 < , ≤ 108	693	0	98.5
108 < , ≤ 120	552	0	98.5
120 < , ≤ 132	449	0	98.5
132 < , ≤ 144	403	0	98.5
144 < , ≤ 156	351	0	98.5
156 < , ≤ 168	253	0	98.5
168 < , ≤ 180	180	2	98.5–97.4
180 < , ≤ 192	68	0	97.4
192 <	8	0	97.4

**Table 5:** Survival rate and number of implants depending on the investigation interval (implant level in the mandible)

Investigation interval (months)	Number of implants	Failed implants	Survival rate intervals (%)
0 < , ≤ 12	1040	3	100–99.7
12 < , ≤ 24	1037	0	99.7
24 < , ≤ 36	1037	1	99.7–99.6
36 < , ≤ 48	1036	0	99.6
48 < , ≤ 60	956	0	99.6
60 < , ≤ 72	899	0	99.6
72 < , ≤ 84	787	1	99.6–99.5
84 < , ≤ 96	707	0	99.5
96 < , ≤ 108	555	0	99.5
108 < , ≤ 120	461	0	99.5
120 < , ≤ 132	381	0	99.5
132 < , ≤ 144	339	2	99.5–98.9
144 < , ≤ 156	301	0	98.9
156 < , ≤ 168	184	0	98.9
168 < , ≤ 180	117	0	98.9
180 < , ≤ 192	49	0	98.9
192 <	4	0	98.9

**Table 6:** Survival rate and number of implants depending on the investigation interval (patient level in the maxilla)

Investigation interval (months)	Number of patients	Failed patients	Survival rate intervals (%)
0 < , ≤ 12	307	8	100–97.4
12 < , ≤ 24	299	1	97.4–97.1
24 < , ≤ 36	298	1	97.1–96.7
36 < , ≤ 48	297	0	96.7
48 < , ≤ 60	282	0	96.7
60 < , ≤ 72	257	0	96.7
72 < , ≤ 84	223	0	96.7
84 < , ≤ 96	189	0	96.7
96 < , ≤ 108	155	0	96.7
108 < , ≤ 120	124	0	96.7
120 < , ≤ 132	100	0	96.7
132 < , ≤ 144	90	0	96.7
144 < , ≤ 156	78	0	96.7
156 < , ≤ 168	58	0	96.7
168 < , ≤ 180	42	1	96.7–94.4
180 < , ≤ 192	16	0	94.4
192 <	5	0	94.4

**Table 7:** Survival rate and number of implants depending on the investigation interval (patient level in the mandible)

Investigation interval (months)	Number of patients	Failed patients	Survival rate intervals (%)
0 < , ≤ 12	254	3	100–98.8
12 < , ≤ 24	251	0	98.8
24 < , ≤ 36	251	1	98.8–98.4
36 < , ≤ 48	250	0	98.4
48 < , ≤ 60	233	0	98.4
60 < , ≤ 72	219	0	98.4
72 < , ≤ 84	191	1	98.4–97.9
84 < , ≤ 96	171	0	97.9
96 < , ≤ 108	133	0	97.9
108 < , ≤ 120	110	0	97.9
120 < , ≤ 132	91	0	97.9
132 < , ≤ 144	81	1	97.9–96.7
144 < , ≤ 156	72	0	96.7
156 < , ≤ 168	45	0	96.7
168 < , ≤ 180	29	0	96.7
180 < , ≤ 192	12	0	96.7
192 <	1	0	96.7

**Fig. 3:**
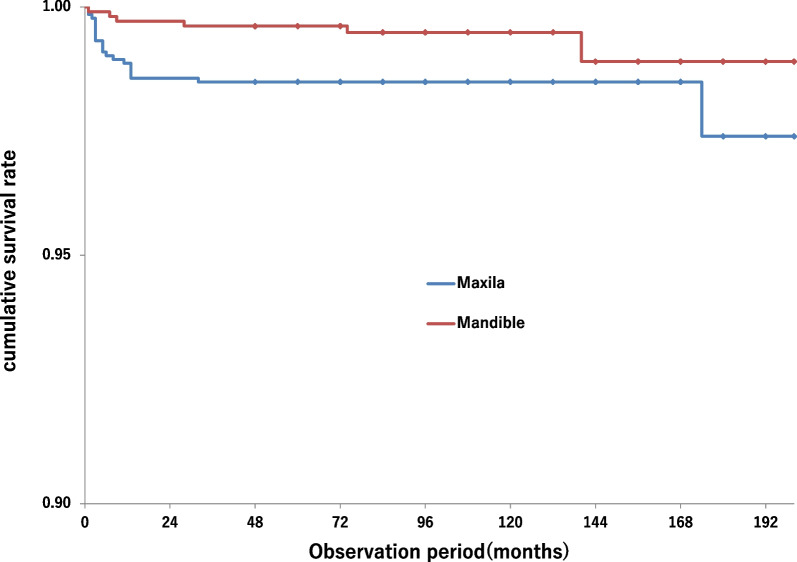
Kaplan–Meier curves in the implant level. The log-rank test showed a significant difference between the maxilla and mandible (*p* = 0.0320)

**Fig. 4:**
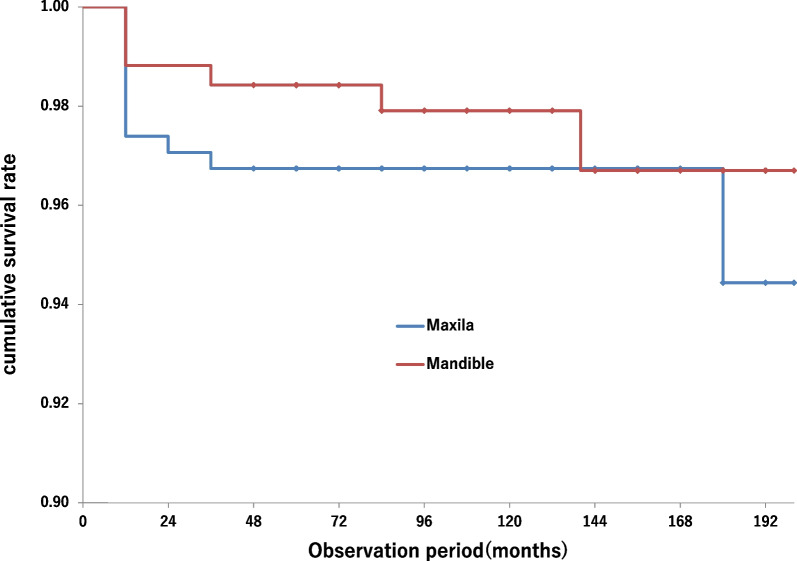
Kaplan–Meier curves in the patient level. The log-rank test showed no significant difference between the maxilla and mandible (*p* = 0.42)

The cumulative implant survival rate over 3–17 years was 94.4% at the patient level and 97.4% at the implant level for the maxilla. For the mandible, the cumulative survival rates were 96.7% at the patient level and 98.9% at the implant level. The cumulative survival rate in the maxilla was significantly lower at the implant level than in the mandible (*p* < 0.05), but not significantly different at the patient level (Figs. 3 and 4).

### Patient profile of implant failure and early implant failure

The details of implant failure cases for the maxilla and mandible are presented in Tables 8 and 9.

**Table 8:** Implant failure case in the maxilla

Case	Age	Sex	Smoking	Systemic disease	Surgical procedure (number of implants)	Type of implant	Diameter (mm)	Length (mm)	Initial torque value (Ncm)	Direction	Time to implant failure (month)
1	56	M	–	–	All-on-four (4)	Nobel speedy groovy	4.0	18	45	Tilted	5
2	51	M	+	–	All-on-four (4)	Nobel replace tapered groovy	4.3	16	45	Straight	3
						Nobel speedy groovy	4.0	18	35	Tilted	3
						Nobel speedy groovy	4.0	18	45	Tilted	3
3	36	M	+	–	All-on-four (4)	Nobel speedy groovy	4.0	18	45	Tilted	2
4	64	F	–	–	All-on-four + 1 (5)	Nobel speedy groovy	4.0	18	30	Tilted	174
						Nobel speedy groovy	4.0	10	35	Straight	174
5	62	M	–	HT	All-on-four (4)	Nobel speedy groovy	4.0	18	50≦	Tilted	8
6	52	F	–	–	All-on-four + 1 (5)	Nobel speedy groovy	4.0	11.5	45	Straight	1
						Nobel speedy groovy	4.0	13	45	Tilted	1
7	66	F	+	–	All-on-four (4)	Nobel speedy groovy	4.0	10	50 ≤	Straight	3
						Nobel speedy groovy	4.0	15	50 ≤	Tilted	6
						Nobel speedy groovy	4.0	10	50 ≤	Straight	3
						Nobel speedy groovy	4.0	15	50 ≤	Tilted	3
8	73	M	–	Hepatitis C	All-on-four (4)	Nobel speedy groovy	4.0	11.5	50 ≤	Straight	11
9	60	M	+	–	All-on-four (4)	Nobel speedy groovy	4.0	13	50 ≤	Straight	13
						Nobel speedy groovy	4.0	18	50 ≤	Tilted	13
						Nobel speedy groovy	4.0	13	50 ≤	Straight	13
						Nobel speedy groovy	4.0	18	50 ≤	Tilted	13
10	60	M	–	HT	All-on-four (4)	Straumann bone level tapered	4.1	18	50 ≤	Tilted	32
11	66	F	–	Arrhythmia	All-on-four + 1 (5)	Nobel parallel CC	3.75	8.5	40	Straight	5
				Osteoporosis		Nobel speedy groovy	4.0	10	45	Straight	5

**Table 9:** Implant failure case in the mandible

Case	Age	Sex	Smoking	Systemic disease	Surgical procedure (number of implants)	Type of implant	Diameter (mm)	Length (mm)	Primary stability (N cm)	Direction	Time to implant failure (month)
1	63	M	+	Stomach cancer	All-on-four (4)	Nobel speedy groovy	4.0	18	45	Tilted	74
2	58	M	+	−	All-on-four (4)	Nobel speedy groovy	4.0	15	35	Tilted	1
3	67	F	−	−	All-on-four + 2 (6)	Nobel speedy groovy	4.0	18	50 ≤	Tilted	140
						Nobel speedy groovy	4.0	7	40	Straight	140
4	55	M	+	Hepatic dysfunction	All-on-four (4)	Nobel speedy groovy	4.0	15	50 ≤	Straight	7
5	38	M	+	DM	All-on-four (4)	Nobel speedy groovy	4.0	15	50 ≤	Tilted	9
6	47	M	+	−	All-on-four (4)	Nobel speedy groovy	4.0	18	50 ≤	Tilted	28

The survival rate was then classified into within 24 months and after 24 months. The maxillary survival rate within 24 months was 97.1% at the patient level and 98.6% at the implant level. After 24 months, the survival rate was 99.3% at the patient level and 99.8% at the implant level (*p* < 0.05 and < 0.01, respectively).

The mandibular survival rate within 24 months was 98.8% at the patient level and 99.7% at the implant level. After 24 months, the survival rate was 98.8% at the patient level and 99.6% at the implant level, with no significant difference (Table 10).

**Table 10:** Time to implant failure

		Patient level (%)	*p* value	Implant level (%)	*p* value
Maxilla					
	≤ 24 months	97.1	0.037*	98.6	0.0014**
	> 24 months	99.3		99.8	
Mandible					
	≤ 24 months	98.8	0.99	99.7	0.99
	> 24 months	98.8		99.6	
Maxilla	≤ 24 months	97.1	0.15	98.6	0.009**
Mandible	≤ 24 months	98.8		99.7	

*p < 0.05, **p < 0.01: Chi-square test

Furthermore, a comparison of the maxilla and mandible regarding implant failure within 24 months resulted in a higher risk of early failure in the maxilla (implant level, *p* < 0.01).

### Risk factors related to survival rate

#### Implant-related factors

##### *Implant type* (Table 11)

**Table 11:** Type of implant to survival rate

	Number of implant placement	Number of implant failure	Survival rate (%)	*p* value
*Maxilla*				
Nobel speedy groovy	1064	18	98.3	–
Straumann bone level tapered	143	1	99.3	0.59
Nobel parallel CC	46	1	97.8	0.73
Nobel replace tapered groovy	38	2	94.7	0.31
Nobel replace tapered	19	0	100	0.56
Brånemark System Mk IV TiUnite	11	0	100	0.66
Nobel active	3	0	100	0.82
*Mandible*				
Nobel speedy groovy	861	7	99.2	–
Straumann bone level tapered	135	0	100	0.29
Brånemark System Mk IV TiUnite	21	0	100	0.68
Nobel replace tapered groovy	12	0	100	0.76
Nobel parallel CC	6	0	100	0.82
Nobel replace tapered	4	0	100	0.86
Nobel active	1	0	100	0.92
*Total*				
Nobel speedy groovy	1925	25	98.7	–
Straumann bone level tapered	278	1	99.6	0.17
Nobel parallel CC	52	1	98.1	0.69
Nobel replace tapered groovy	50	2	96.0	0.43
Brånemark System Mk IV TiUnite	32	0	100	0.51
Nobel replace tapered	23	0	100	0.58
Nobel active	4	0	100	0.82

*p < 0.05, **p < 0.01: Chi-square test

Regarding differences in survival rate according to implant type, the Nobel Speedy Groovy implant (Nobel Biocare AG, Kloten, Switzerland) had survival rates of 98.3% and 99.2% for maxillary and mandibular implants, respectively; the Bone Level Tapered implant (Straumann AG, Basel, Switzerland) had survival rates of 99.3% and 100% for maxillary and mandibular implants, respectively; the Nobel Parallel CC implant (Nobel Biocare AG, Kloten, Switzerland) had survival rates of 97.8% and 100% for maxillary and mandibular implants, respectively; and the Nobel Replace Tapered Groovy implant had survival rates of 94.7% and 100% for maxillary and mandibular implants, respectively. No significant differences were observed among the survival rates according to implant type for either the maxilla or the mandible.

##### Implant length (Table 12)

**Table 12:** Implant length to survival rate

	Number of implant placed	Number of implant failure	Survival rate (%)	*p* value
*Maxilla*				
< 10 mm	37	1	97.3	–
10 mm ≤ , < 15 mm	409	9	97.8	0.70
15 mm ≤ , < 18 mm	342	3	99.1	0.85
18 mm ≤	536	9	98.3	0.85
*Mandible*				
< 10 mm	17	1	94.1	–
10 mm ≤ , < 15 mm	206	0	100	0.10
15 mm ≤ , < 18 mm	357	3	99.2	0.048*
18 mm ≤	460	3	99.3	0.022*
*Total*				
< 10 mm	54	2	96.3	–
10 mm ≤ , < 15 mm	615	9	98.5	0.21
15 mm ≤ , < 18 mm	699	6	99.1	0.049*
18 mm ≤	996	12	99.3	0.12

*p < 0.05, **p < 0.01: Chi-square test

Regarding the differences in survival rate according to implant length, implants of < 10 mm in length had survival rates of 97.3% and 94.1% for maxillary and mandibular implants, respectively; implants of 10 mm ≤ , < 15 mm had survival rates of 97.8% and 100% for maxillary and mandibular implants, respectively; implants of 15 mm ≤ , < 18 mm had survival rates of 99.1% and 99.2% for maxillary and mandibular implants, respectively; and implants of 18 mm ≤ had survival rates of 98.3% and 99.3% for maxillary and mandibular implants, respectively. Implant lengths < 15 mm showed significantly higher survival rates in the mandible (*p* < 0.05), but no significant differences were observed in the maxilla.

##### Primary stability (Table 13)

**Table 13:** Primary stability to survival rate

	Number of implant placed	Number of implant failure	Survival rate (%)	*p* value
*Maxilla*				
< 35 Ncm	113	1	99.1	–
35 Ncm ≤ , < 50 Ncm	334	10	97.0	0.37
50 Ncm ≤	877	11	98.7	0.91
*Mandible*				
< 35 Ncm	31	0	100	–
35 Ncm ≤ , < 50 Ncm	248	3	98.8	0.76
50 Ncm ≤	761	4	99.5	0.37
*Total*				
< 35 Ncm	144	1	99.3	–
35 Ncm ≤ , < 50 Ncm	582	13	97.8	0.23
50 Ncm ≤	1638	15	99.1	0.78

*p < 0.05, **p < 0.01: Chi-square test

The survival rates according to primary stability at implantation are as follows: implants with a value of < 35 Ncm had survival rates of 99.1% and 100% for the maxilla and mandible, respectively; implants with a value of 35–50 Ncm had survival rates of 97.0% and 98.8% for maxillary and mandibular implants, respectively; and implants with a value of > 50 Ncm had survival rates of 98.7% and 99.5% for maxillary and mandibular implants, respectively. No significant differences were observed in the survival rates according to primary stability for either the maxilla or the mandible.

##### Angle of implant placement (Table 14)

**Table 14:** Angle of implant placement to survival rate

	Number of implant placed	Number of implant failure	Survival rate (%)	*p* value
*Maxilla*				
Straight	710	10	98.6	0.57
Tilted	614	12	98.0	
*Mandible*				
Straight	532	2	99.6	0.41
Tilted	508	5	99.0	
*Total*				
Straight	1242	12	99.0	0.22
Tilted	1122	17	98.5	

*p < 0.05, **p < 0.01: Chi-square test

The survival rates of implants placed in the axial direction were 98.6% and 99.6% for the maxilla and mandible, respectively. The survival rates of tilted implants were 98.0% and 99.0% for the maxilla and mandible, respectively. No significant difference in survival rates due to the placement angle of the implant was observed for either the maxilla or the mandible.

#### Patient-related factors

##### *Sex* (Table 15)

**Table 15:** Sex to survival rate

	Number of patients	Number of implants	Patient level (number of patient failure)	*p* value	Implant level (number of implant failure)	*p* value
*Maxilla*						
Male	156	658	95.5% (7)	0.58	98.2% (12)	0.81
Female	151	666	97.4% (4)		98.5% (10)	
*Mandible*						
Male	131	532	96.2% (5)	0.25	99.1% (5)	0.48
Female	123	508	99.2% (1)		99.6% (2)	
*Total*						
Male	287	1190	95.8% (12)	0.11	98.6% (17)	0.36
Female	274	1174	98.2% (5)		99.0% (12)	

*p < 0.05, **p < 0.01: Chi-square test

For the maxilla, male patients had survival rates of 95.5% at the patient level and 98.2% at the implant level. Female patients had rates of 97.4% at the patient level and 98.5% at the implant level.

For the mandible, male patients had survival rates of 96.2% at the patient level and 99.1% at the implant level. Female patients had survival rates of 99.2% at the patient level and 99.6% at the implant level.

No significant differences were observed for either the maxilla or the mandible.

##### *Systemic disease* (Table 16)

**Table 16:** Systemic disease to survival rate

	Number of patients	Number of implant placed	Patient level (number of patient failure)	*p* value	Implant level (number of implant failure)	*p* value
*Maxilla*						
Presence	130	564	96.9% (4)	0.92	99.1% (5)	0.09
Absence	177	760	96.0% (7)		97.8% (17)	
*Mandible*						
Presence	99	408	97.0% (3)	0.89	99.3% (3)	0.84
Absence	155	632	98.1% (3)		99.4% (4)	
*Total*						
Presence	229	972	96.9% (7)	0.97	99.2% (8)	0.14
Absence	332	1392	97.0% (10)		98.5% (21)	

*p < 0.05, **p < 0.01: Chi-square test

In this study, we investigated the survival rate of implants with diabetes mellitus [10, 11], cardiovascular disease [10, 12, 13], and osteoporosis [14], which have been reported as causes of implant failure and peri-implantitis, as the main systemic diseases.

For the maxilla, healthy participants had survival rates of 96.0% at the patient level and 97.8% at the implant level. Patients with systemic disease had survival rates of 96.9% at the patient level and 99.1% at the implant level.

For the mandible, healthy participants had survival rates of 98.1% at the patient level and 99.4% at the implant level. Patients with systemic disease had survival rates of 97.0% at the patient level and 99.3% at the implant level. No significant differences were observed for either the maxilla or the mandible.

##### *Smoking* (Table 17)

**Table 17:** Smoking to survival rate

	Number of patients	Number of implant placed	Patient level (number of patient failure)	*p* value	Implant level (number of implant failure)	*p* value
*Maxilla*						
Smoker	104	446	96.2% (4)	0.86	97.3% (12)	0.037*
Non smoker	203	878	96.6% (7)		98.9% (10)	
*Mandible*						
Smoker	96	392	94.8% (5)	0.019*	98.7% (5)	0.065
Non smoker	158	648	99.4% (1)		99.7% (2)	
*Total*						
Smoker	200	838	95.5% (9)	0.13	97.9% (17)	0.0086**
Non smoker	361	1526	97.8% (8)		99.2% (12)	

*p < 0.05, **p < 0.01: Chi-square test

For both the maxilla and mandible, smokers had implant survival rates of 95.5% at the patient level and 97.9% at the implant level, whereas non-smokers had implant survival rates of 97.8% at the patient level and 99.2% at the implant level. Survival rates at the implant level were significantly lower for smokers (*p* = 0.0086 < 0.01).

For the maxilla, smokers had implant survival rates of 96.2% at the patient level and 97.3% at the implant level, whereas non-smokers had implant survival rates of 96.6% at the patient level and 98.9% at the implant level. Smokers had significantly lower survival rates than non-smokers at the implant level (*p* < 0.05).

For the mandible, smokers had implant survival rates of 94.8% at the patient level and 98.7% at the implant level, whereas non-smokers had implant survival rates of 99.4% at the patient level and 99.7% at the implant level. Smokers had significantly lower survival rates than non-smokers at the patient level (*p* < 0.05).

### Risk factors for survival rate multivariate analysis

The risk factors influencing the survival rate are shown in Table 18. To identify the risk factors for survival rate (implant failure), logistic regression analyses were performed at both the implant and patient levels. The factor with the greatest influence on survival was the maxilla in the treatment area, with an OR of 1.98 at the patient level and 5.68 at the implant level, which was significantly different (*p* = 0.0034, implant level). However, smoking was not statistically significant, although the OR was 1.98 at the patient level and 2.58 at the implant level (*p* = 0.39, *p* = 0.37, respectively).

**Table 18:** Risk factors for survival rate multivariate analysis (odds ratio)

	Risk factor	Odds ratio	*p* value
*Implant-related factors*			
Implant length	< 10 mm	1.97	> 0.05
Primary stability	< 35 Ncm	0.54	> 0.05
Angle of implant placement	Tilted	1.57	> 0.05
*Patient-related factors for patient-level*			
Treatment area	Maxilla	1.54	> 0.05
Sex	Male	1.40	> 0.05
Systemic disease	Presence	1.01	> 0.05
Smoking	Smoking	1.98	> 0.05
*Patient-related factors for implant-level*			
Treatment area	Maxilla	5.68	< 0.01**
Sex	Male	2.35	> 0.05
Systemic disease	Presence	0.51	> 0.05
Smoking	Smoking	2.58	> 0.05

*p < 0.05, **p < 0.01: logistic regression analysis

## Discussion

### Cumulative implant survival rate for full-arch rehabilitation

In this study, the survival rate of implants after ≥ 3 years was 96.4% at the patient level and 98.3% at the implant level for the maxilla, and 98.1% at the patient level and 99.3% at the implant level for the mandible.

In a systematic review [15] of the survival rate of implants in fixed prosthetic treatment with implants for edentulous cases, under the conventional loading protocol, the maxillary survival rate was 94.95–100% (2–15-year follow-up), and the mandibular survival rate was 96.47–100% (3–15-year follow-up). The early loading protocol had survival rates of 94.7–100% for maxillary implants (1–3-year follow-up) and 98.51–100% for mandibular implants (1–2-year follow-up). Finally, under the immediate loading protocol, the survival rates were 90.43–100% (follow-up period: 1–10 years) for the maxilla and 90–100% (follow-up period: 1–10 years) for the mandible. The survival rate of implants in treatment based on the All-on-four concept was reported to be 94.7% (5–13 years) in the maxilla and 93% (10–18 years) in the mandible at the implant level [3, 4]. In the latest review, it was reported to be 93.9–100% for the maxilla (up to 13 years) and 91.7–100% for the mandible (up to 18 years) [16]. Furthermore, in a recent systematic review, the mean cumulative residuals over 72–132 months were as high as 94–98% [17]. The results of the present study are consistent with those of previous studies, including systematic reviews, and suggest that the All-on-four concept may be a beneficial treatment method in the Japanese population.

Some studies reported [18, 19] no significant difference between the survival rates of maxillary and mandibular implants in treatment based on the All-on-four concept. However, in the present study, similar to the report by Ping et al. [20] (maxilla: 92.8%, mandible: 99.0%, mean follow-up period: 2.8 years), the survival rate of implants in the maxilla was significantly lower than that of implants in the mandible at the implant level. The findings of the present study differed from those of Malo et al. probably because of the different observation periods and the influence of skeletal patterns and bone quality due to differences in the race of the patients.

Multiple factors are involved in implant failure, and, in accordance with their timing, we can divide failure occurrences into early and late failures. Early failure is primarily due to failure to achieve osseointegration. Late failure is said to be caused by a bacterial infection or excessive burden after achieving osseointegration. Failures that occur prior to the final superstructure installation are classified as early failures. Those that occur after installation (loading) are classified as late failures. However, some studies have classified the period within 1 year after the final superstructure installation as the early period and the period after 1 year as the late period [21, 22]. This classification cannot be applied in the present study, because immediate loading treatment was performed. Therefore, in the present study, we examined the difference in survival rate using the timepoint of 24 months as a reference point.

In the present study, there were 22 implant failures in 11 cases in the maxilla and seven failures in six cases in the mandible. In particular, for maxillary implants, 19 implants (86.4%) in nine cases failed early, i.e., within 24 months (1–13 months) of implantation. That is, the survival rate within 24 months was significantly low at both the patient and implant levels. Maló et al. [14] reported implant failure in 19 of 968 implants after 5 years in maxillary cases treated based on the All-on-four concept. Among those 19, 16 (84.2%) failed within 12 months after implantation. That is, a trend similar to that of the present study was observed. In addition, there was a tendency for multiple maxillary implant failures per patient, with an average of 2.1 per patient. This tendency is called cluster failures [13, 23] (a phenomenon in which failures of implant treatment are concentrated in a certain group of patients rather than occurring uniformly in all patients). Implant-related factors include mechanically polished surface texture and short implants. Patient-related factors include age, poor bone quality, oral proton pump inhibitor use, smoking, and bruxism. In the present study, we believe that bone quality was not the only reason for the high rate of early failure of maxillary implants. Since immediate loading was performed, it is thought that the effects of opposing teeth and bruxism may have also been involved.

### Factors related to survival rate

#### Implant-related factors

In the present study, there was no significant difference in the survival rate between axial and tilted implants in either the maxilla or the mandible. A recent systematic review [17] reported no significant difference in the survival rate and marginal bone loss between axial and tilted implants placed during treatment using the All-on-four concept. One advantage of tilted placement is that it enables the placement of a longer implant compared to placement in the axial direction. As a result, the contact area between the implant and the bone increases, and the possibility of obtaining better primary stability increases [22]. Zampelis et al. [24] performed finite-element analysis on the difference in the stress applied to the bone around the implant site when the implant was placed in the axial direction, when the implant was placed tilted, and with or without a cantilever. They reported no difference in stress between axial and tilted implants, and the difference was dependent on the presence or absence of the cantilever. It is biomechanically advantageous to shorten the distance of the cantilever via tilted implantation. Therefore, there is no difference in the amount of marginal bone resorption and the survival rate of the implant between the angled placement using this technique and the placement along the tooth axis. Furthermore, since the cantilever can be shortened by embedding in the centrifugal slope, it is considered to be a superior method in terms of mechanics.

Regarding the effects of length and primary stability, the consensus statement [25] on fixed prosthetic treatment with implants in edentulous maxillary patients recommends the placement of at least four implants. Furthermore, to perform immediate loading, it is recommended to place an implant with a length of ≥ 10 mm and obtain primary stability of ≥ 30 Ncm. In the present study, in all cases (34 cases) in which an implant with a length of ≥ 10 mm could not be placed, additional implants were placed nearby. Regarding primary stability, the All-on-four concept consensus statement [26] recommends achieving a primary stability of ≥ 35 Ncm. Further reports [27, 28] recommend the acquisition of primary stability of 35 Ncm when performing immediate loading. In the present study, additional implantation was performed, where it is possible (57 cases = 78.1%) for cases (73 cases) in which primary stability of ≥ 35 Ncm could not be achieved. In the present study, there was no significant difference in survival rate due to implant length or primary stability. However, one reason for this was likely that the load could be distributed by performing additional insertions near implants that did not meet the criteria. There was no significant difference in the length of the implant and the survival rate, but the survival rate was low for implants < 10 mm in both the maxilla and mandible. Indeed, the OR indicated a 1.97-fold increased risk of failure for implants < 10 mm in length. In addition to situations in which implants with a length of < 10 mm must be placed due to problems with residual bone height, if sufficient contact area with the bone cannot be obtained, such as when the thread is exposed in the extraction socket, additional implantation should be performed nearby. That is, it is important to attempt to disperse the load by doing so. With respect to primary stability, in maxillary cases treated with the All-on-four concept, there was no difference in the survival rate of implants with primary stability > 30 or < 30 Ncm, even without additional placement [29]. It is also reported that linked implants can be loaded immediately even if the primary stability value is < 20 Ncm [30]. Thus, it is necessary to further examine the criteria for primary stability values that permit immediate loading.

#### Patient-related factors

In the present study, we investigated the survival rate of implants in relation to the presence or absence of systemic disease. We did not observe any significant differences in survival rate between the presence or absence of systemic diseases for either the maxilla or the mandible. However, due to the small number of patients with each disease, it is not possible to draw conclusions from these results alone. There are several reports that patients with poorly controlled diabetes mellitus [10, 11] and cardiovascular disease [10, 12, 13] have a high risk of developing peri-implantitis and failure. Furthermore, it has been reported that patients receiving bisphosphonates for osteoporosis are at risk of implant failure [14]; thus, this is an issue for future investigation.

In the present study, a significant decrease in implant survival rate was observed in smokers in both the maxilla and mandible in univariate analysis. Similarly, there are several reports that smoking reduces the survival rate of implants [18, 31–33], and Maló et al. [33] reported that the survival rate of implants placed in smokers continued to decline after 5 and 10 years. Long-term follow-up and smoking cessation guidance are considered necessary for smokers undergoing implant treatment.

In the present study, there were no significant differences in implant survival by sex in both the maxilla and mandible. However, Malo et al. reported an association between male sex and decreased implant survival and increased marginal bone loss in the maxilla [3] and an association between male sex and increased mechanical complications, such as superstructure and screw fracture in the mandible [4], suggesting that more careful follow-up is considered necessary.

This was a limited-scope study that did not examine factors, such as the causes of tooth loss, the condition of opposing teeth (edentulous or dentulous), the presence or absence of bruxism, or multiple comparisons of risk factors. In particular, there are several studies indicating that bruxism is an important patient-related risk factor. De Angelis et al. [34] compared four localized risk factors—bruxism, smoking, bone augmentation, and lateral load on the implant body—and reported that bruxism was the greatest risk factor. Thus, because bruxism is considered to be a very important factor, we would like to accumulate further data on this and examine its effects in the future.

## Conclusion

We treated the maxillae and mandibles of patients based on the All-on-four concept and studied patients who had undergone treatment ≥ 3 years prior. We obtained the following results:

1. While the implant survival rate after ≥ 3 years was generally high for both the maxilla and mandible, the maxilla had a significantly lower survival rate at the implant level.
2. The survival rates of maxillary implants within 24 months of implantation were significantly low at both the patient and implant levels.
3. The various risk factors influencing the survival rate were examined, with the maxilla of the ‘treatment area’ being the most influential factor.

## Data Availability

All data generated or analysed during this study are included in this published article.

## Abbreviations

OR: Odds ratio
SD: Standard deviation
